# Supplementary material for: Overexpression of mitochondrial fission or mitochondrial fusion genes enhances resilience and extends longevity
Source: Aging Cell. 2024 Jul 2;23(10):e14262. doi: 10.1111/acel.14262 (PMC11464124; doi:10.1111/acel.14262)
Supplement: Supplementary file 1 — Appendix S1. [file ACEL-23-e14262-s001.pdf]

Supplemental Figures and Supplemental Table S1

for

Overexpression of mitochondrial fission or mitochondrial fusion genes enhances resilience and extends longevity

Annika Traa, Allison Keil, Abdelrahman AlOkda, Suleima Jacob-Tomas,
Aura A. Tamez González, Shusen Zhu, Zenith Rudich, Jeremy M. Van Raamsdonk

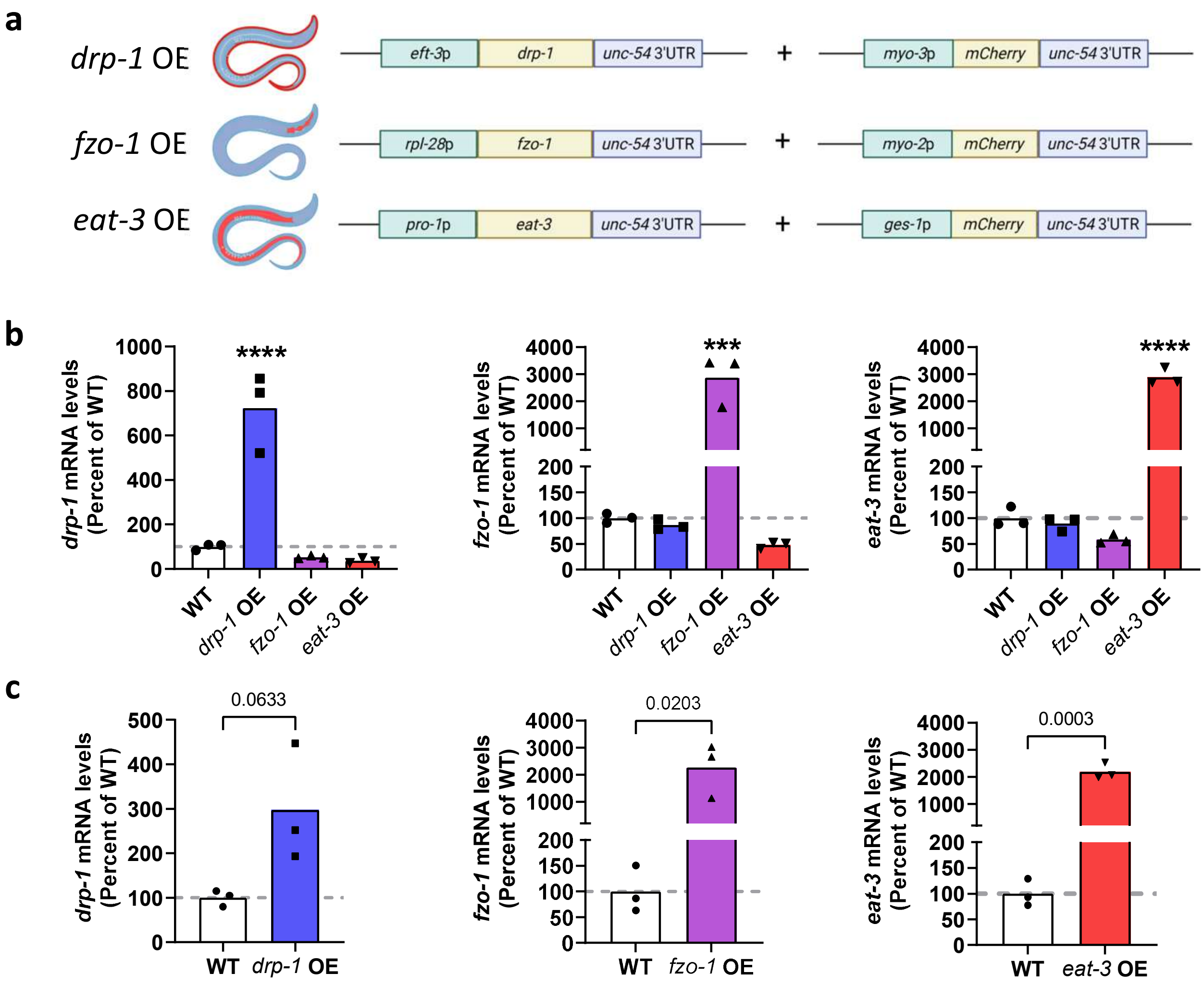

Figure S1. Overexpression of mitochondrial fission or fusion genes in *C. elegans*. *C. elegans* strains that overexpress mitochondrial fission or fusion genes were generated by microinjection and integration of the extrachromosomal array. (**a**) Diagram of constructs used to generate overexpression strains. The mitochondrial fission and fusion genes *drp-1, fzo-1* and *eat-3* were overexpressed under the ubiquitous promoters *eft-3, rpl-28* and *pro-1,* respectively (indicated by blue colouring in worm diagram). Each strain also expressed a red fluorescent co-injection marker in body wall muscle (*drp-1* OE), pharynx (*fzo-1* OE) or intestine (*eat-3* OE). (**b**) Quantitative RT-PCR confirmed that each overexpression strain exhibits increased expression of the intended mitochondrial fission or fusion gene. (**c**) At day 8 of adulthood, the mitochondrial fission or fusion genes remain overexpressed. Three biological replicates were performed. Statistical significance was assessed using a one-way ANOVA with Dunnett's multiple comparisons test in panel b or a t-test in panel c. OE = overexpression. p-values indicate differences from wild-type. ***p<0.001, ****p<0.0001.

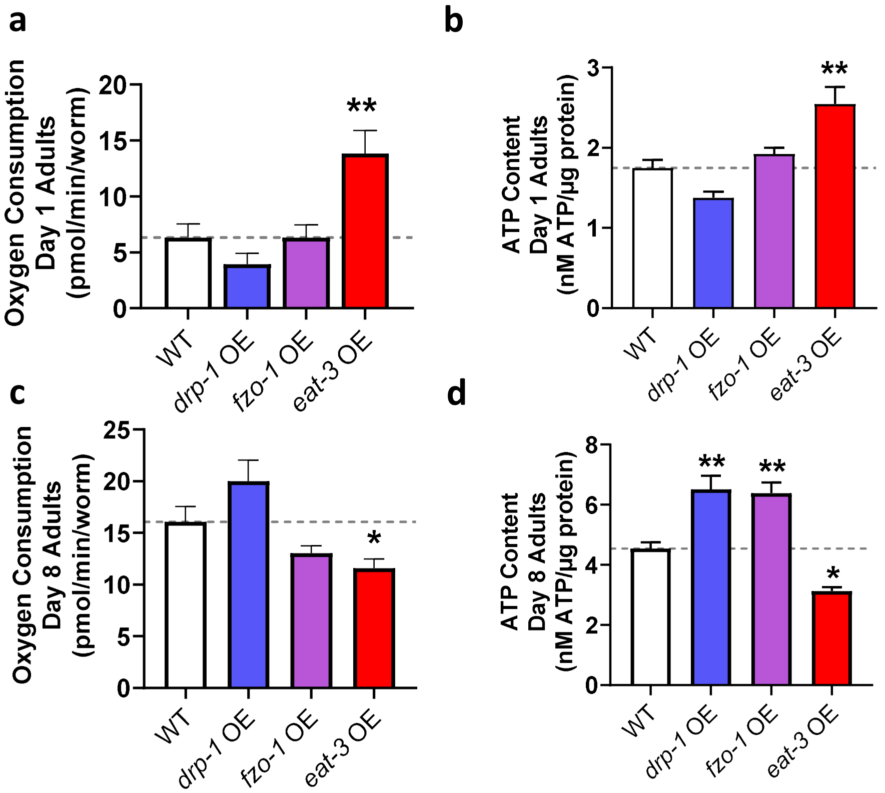

Figure S2. Overexpression of mitochondrial fission or fusion genes alters mitochondrial function. At day 1 of adulthood, *eat-3* OE worms have increased oxygen consumption (**a**) and increased levels of ATP (**b**). In contrast, at day 8 of adulthood, *eat-3* OE worms have decreased oxygen consumption (**c**) and decreased ATP levels (**d**). Error bars indicate SEM. Six biological replicates were performed for oxygen consumption and three biological replicates were performed for ATP measurements. Statistical significance was assessed using a one-way ANOVA with Dunnett's multiple comparisons test. OE = overexpression. p-values indicate differences from wild-type. *p<0.05, **p<0.01, ***p<0.001, ****p<0.0001.

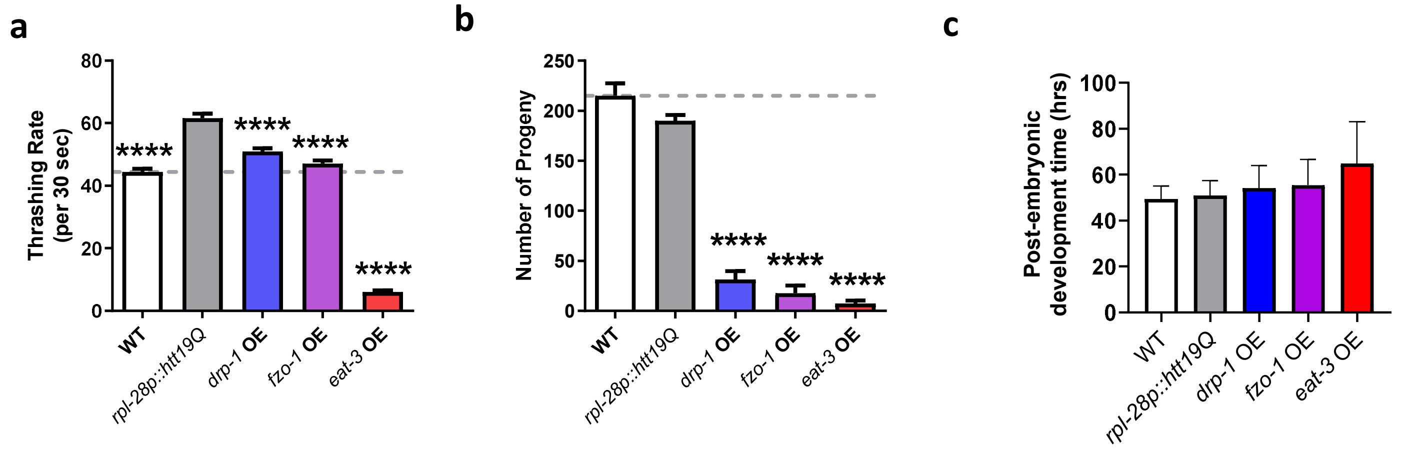

Figure S3. Overexpression of a control protein from *rpl-28* promoter does not recapitulate decreased thrashing or reduced fertility in strains overexpressing mitochondrial fusion genes. *rpl-28p::htt19Q* control worms did not have decreased movement (**a**) or decreased fertility (**b**) compared to wild-type worms. These worms also exhibited wild-type post-embryonic development (**c**). Error bars indicate SEM. A minimum of three biological replicates were performed. Statistical significance was assessed using a one-way ANOVA with Dunnett's multiple comparisons test. OE = overexpression. Data on overexpression strains is also shown in Figures 2. The data on the overexpression strains shown here was collected in the same experiment with wild-type and *rpl-28p::htt19Q* control worms. p-values indicate differences from *rpl-28p::htt19Q* control worms. ****$p<0.0001$.

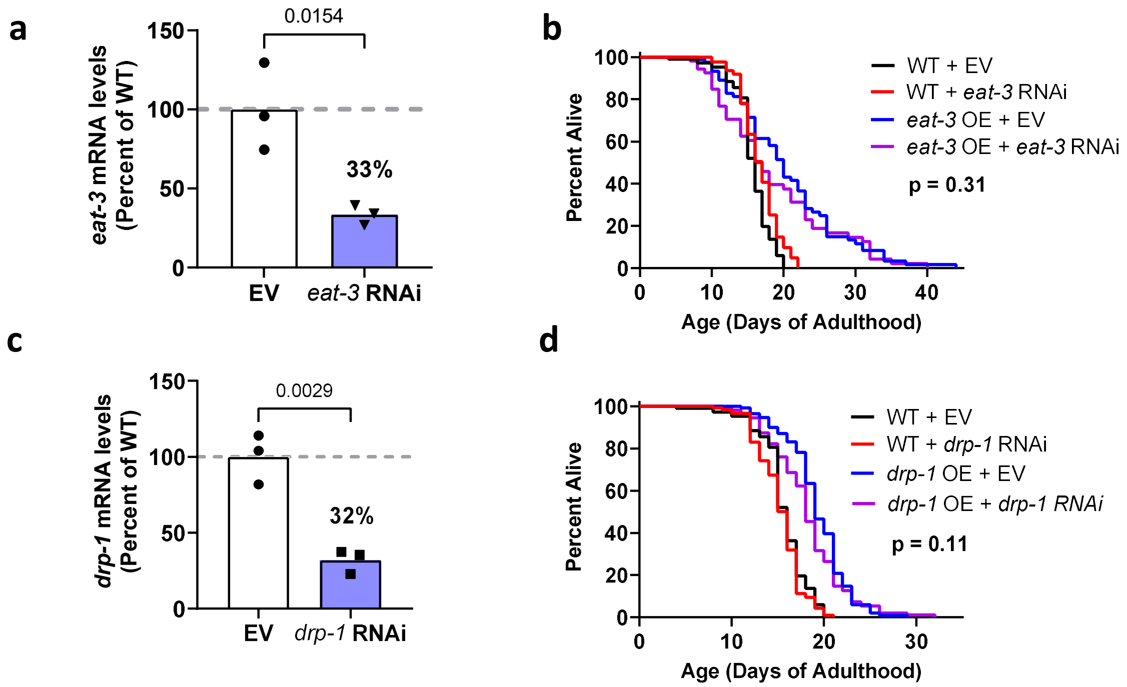

Figure S4. RNA interference decreases the magnitude of mitochondrial fission or fusion gene overexpression but not enough to affect lifespan. (a) *eat-3* RNAi decreases *eat-3* mRNA levels by 67% in *eat-3* OE worms. However, *eat-3* RNAi-treated *eat-3* OE worms still have markedly increased expression of *eat-3* mRNA (almost 10-fold increased from wild-type worms). **(b)** Decreasing *eat-3* expression in *eat-3* OE worms does not affect longevity indicating that 10-fold increase in expression is sufficient to increase lifespan. **(c)** *drp-1* RNAi decreased *drp-1* mRNA by 68%. **(d)** Reducing *drp-1* expression in *drp-1* OE worms does not significantly decrease lifespan. Three biological replicates were performed. Statistical significance was assessed using a t-test in panels a and c, and the log-rank test in panels b and d. p-values in b and d indicate differences between EV and *drp-1* RNAi in *drp-1* OE worms.

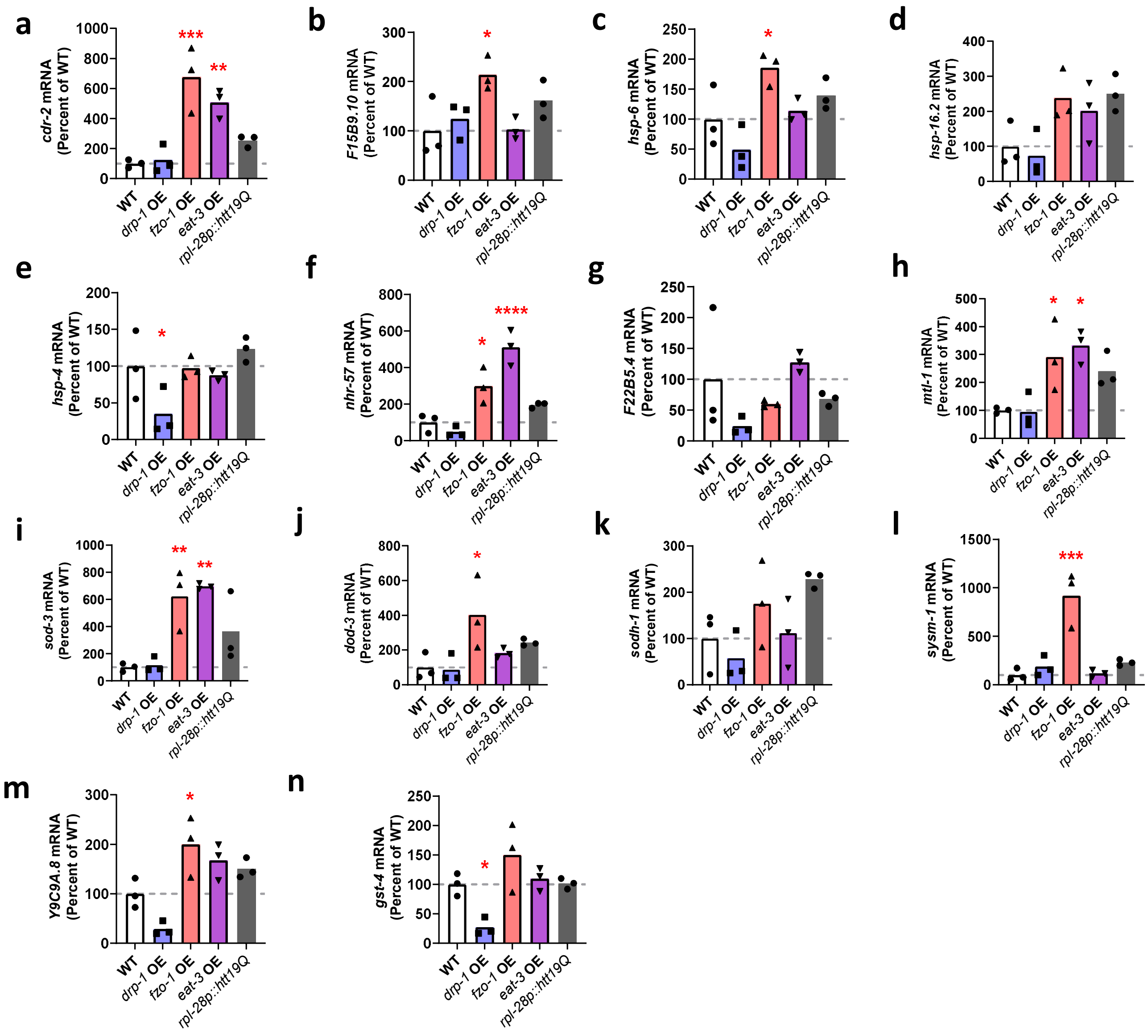

Figure S5. Multiple pathways of cellular resilience are activated at day 8 of adulthood in worms overexpressing mitochondrial fusion genes. Quantitative real-time PCR was used to assess the expression of target genes from different pathways of cellular resilience in the mitochondrial fission and fusion overexpression strains (*drp-1* OE, *fzo-1* OE and *eat-3* OE). As a control, expression was also examined in worms overexpressing a control protein under the *rpl-28* promoter, which was used in the *fzo-1* OE strain. We examined target genes from the mitochondrial unfolded protein response (*cdr-2, F15B9.10, hsp-6;* **a-c**), the cytoplasmic unfolded protein response (*hsp-16.2;* **d**), the ER-unfolded protein response (*hsp-4*; **e**), the hypoxia response (*nhr-57, F22B4.5*; **f,g**), the DAF-16-mediated stress response (*mtl-1, sod-3, dod-3, sodh-1*; **h-k**), the p38-mediated innate immune signaling pathway (*sysm-1, Y9C9A.8*; **l,m**) and the SKN-1-mediated oxidative stress response (*gst-4*; **n**). Similar to day 1 of adulthood, *eat-3* OE and *fzo-1* OE worms show a significant upregulation of target genes of some pathways of cellular resilience, while *drp-1* OE animals do not exhibit any evidence of stress pathway activation. Three biological replicates were performed. Statistical significance was assessed using a one-way ANOVA with Dunnett's multiple comparisons test. p-values indicate differences from wild-type. *p<0.05, **p<0.01, ***p<0.001, ****p<0.0001.

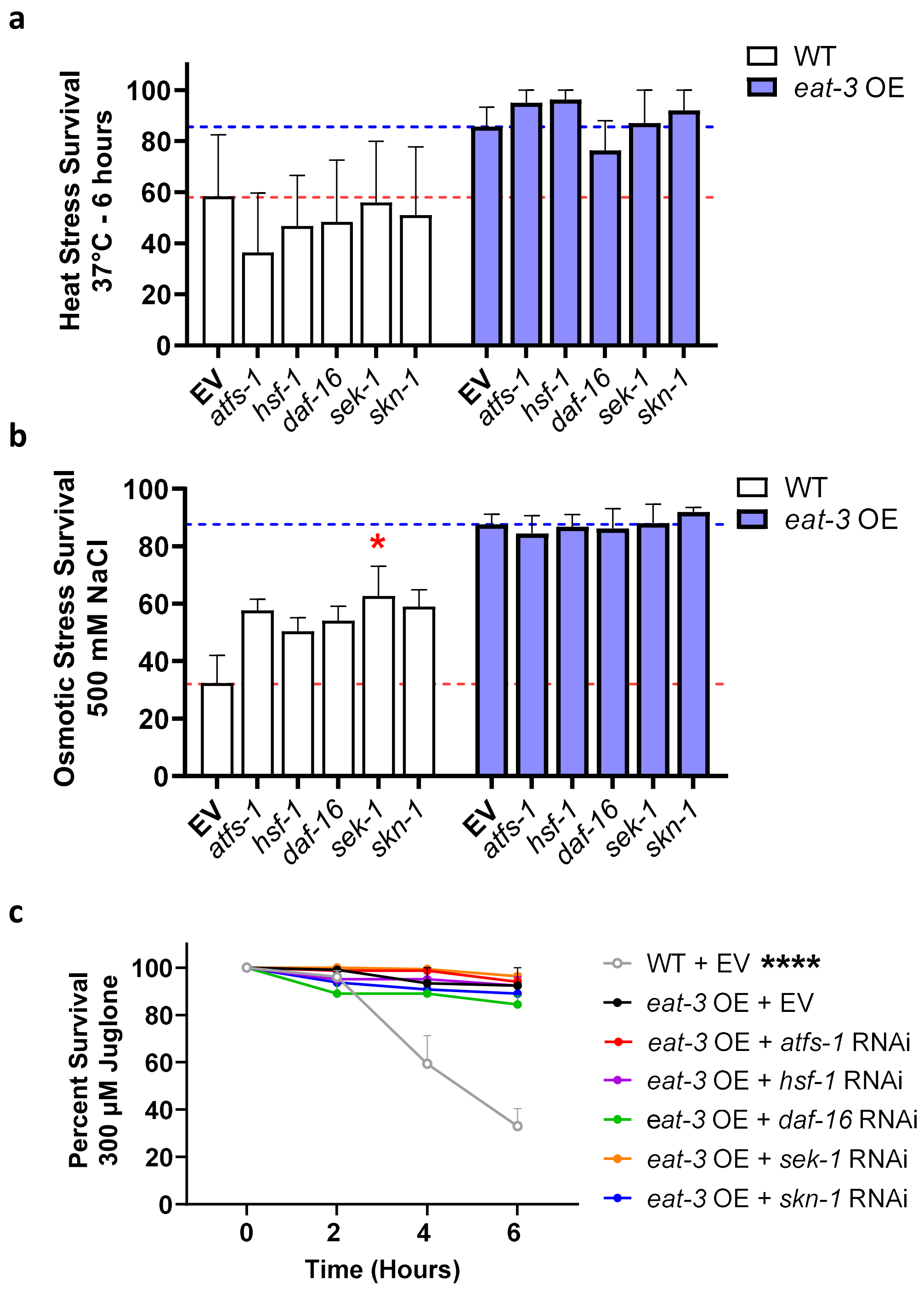

Figure S6. Disruption of pathways of cellular resilience does not decrease stress resistance in *eat-3* OE worms. To assess the contribution of different pathways of cellular resilience to the enhanced stress resistance resulting from the overexpression of the mitochondrial fusion gene *eat-3*, the transcription factors or kinases that mediate these pathways were knocked down using RNAi and stress resistance was measured. We examined resistance to heat stress at 37°C (**a**), osmotic stress at 500 mM (**b**), and oxidative stress through exposure to 300 µM juglone (**c**). In each case, we confirmed that *eat-3* OE animals have enhanced stress resistance compared to wild-type worms. However, disruption of different pathways of cellular resilience by RNAi did not decrease the resistance of *eat-3* OE worms to any of the three exogenous stressors. Three biological replicates were performed. Statistical significance was assessed using a two-way ANOVA with Tukey's multiple comparisons test in panels a and b and a two-way ANOVA with Dunnett's multiple comparisons test in panel c. p-values indicate differences from EV in panels a and b, and differences from *eat-3* OE + EV in panel c. *p<0.05, **** p<0.001.

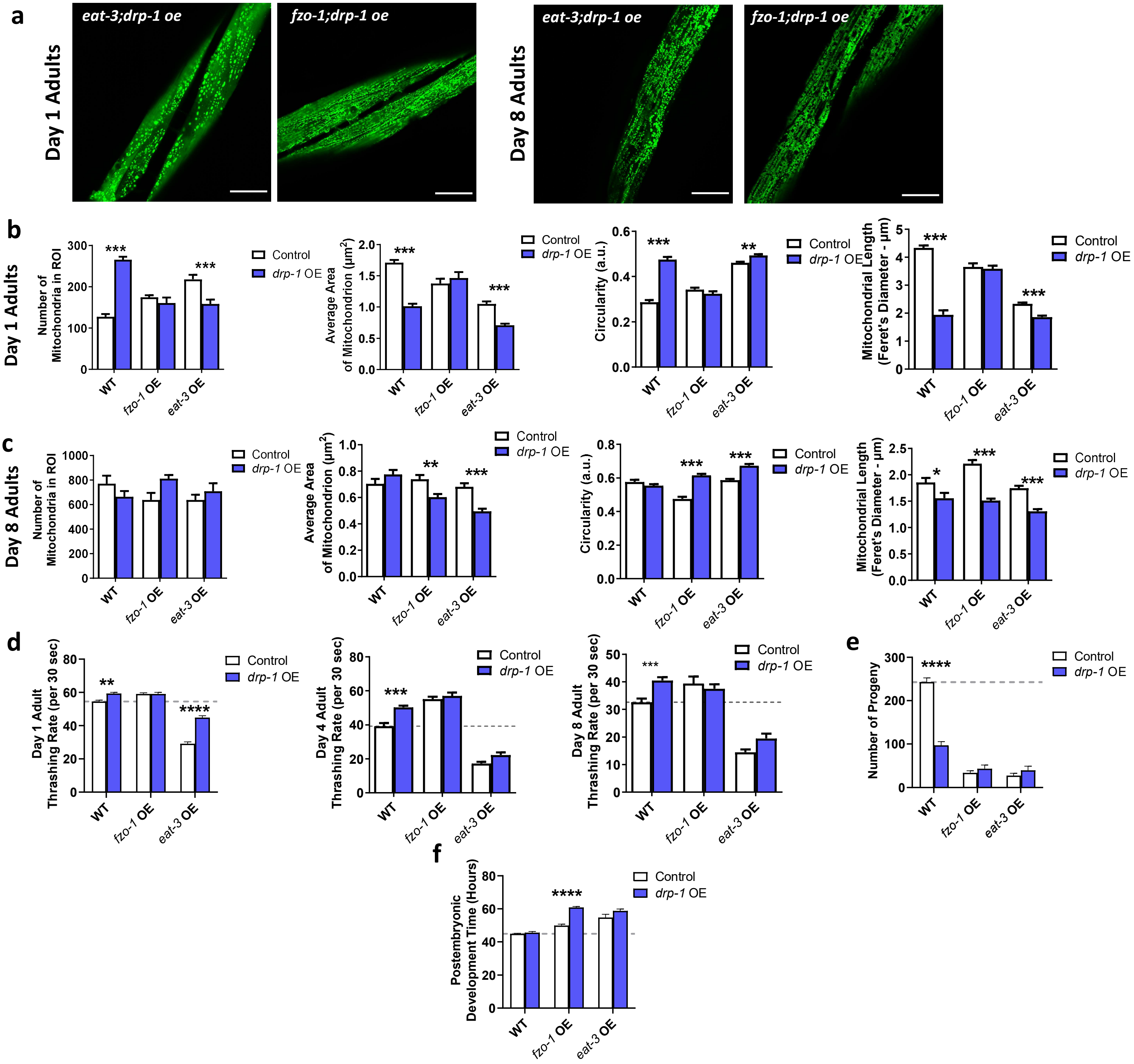

Figure S7. Overexpression *drp-1* causes further mitochondrial fragmentation in *eat-3* OE worms. To determine if overexpression of the mitochondrial fission *drp-1* would diminish phenotypes caused by overexpression of mitochondrial fusion genes, *drp-1* OE worms were crossed with *fzo-1* OE and *eat-3* OE worms. (**a**) Images of mitochondrial morphology in *eat-3* OE*;drp-1* OE and *fzo-1* OE*;drp-1* OE worms at day 1 and day 8 of adulthood. Scale bar indicates 25 µm. (**b**) At day 1 of adulthood, overexpression of *drp-1* decreased mitochondrial number, decreased mitochondrial area, increased mitochondrial circularity and decreased mitochondrial length in *eat-3* OE worms. *drp-1* OE did not affect the mitochondrial morphology of *fzo-1* OE worms. (**c**) At day 8 of adulthood, overexpression of *drp-1* decreased mitochondrial area, increase mitochondrial circularity and decreased mitochondrial length in both *fzo-1* OE and *eat-3* OE worms.(**d**) *drp-1* OE ameliorated the decreased movement of *eat-3* OE worms on day 1 of adulthood but not at day 4 or day 8. (**e**) Overexpression of *drp-1* did not affect fertility in *fzo-1* OE or *eat-3* OE worms and resulted in a slowing of post-embryonic development time in *fzo-1* OE worms (**f**). Errors bars indicate SEM. A minimum of three biological replicates were performed. Statistical significance was assessed using a two-way ANOVA with Šidák's multiple comparisons test. OE = overexpression. p-values indicate differences between control and *drp-1* OE. *p<0.05, **p<0.01, ***p<0.001, ****p<0.0001.

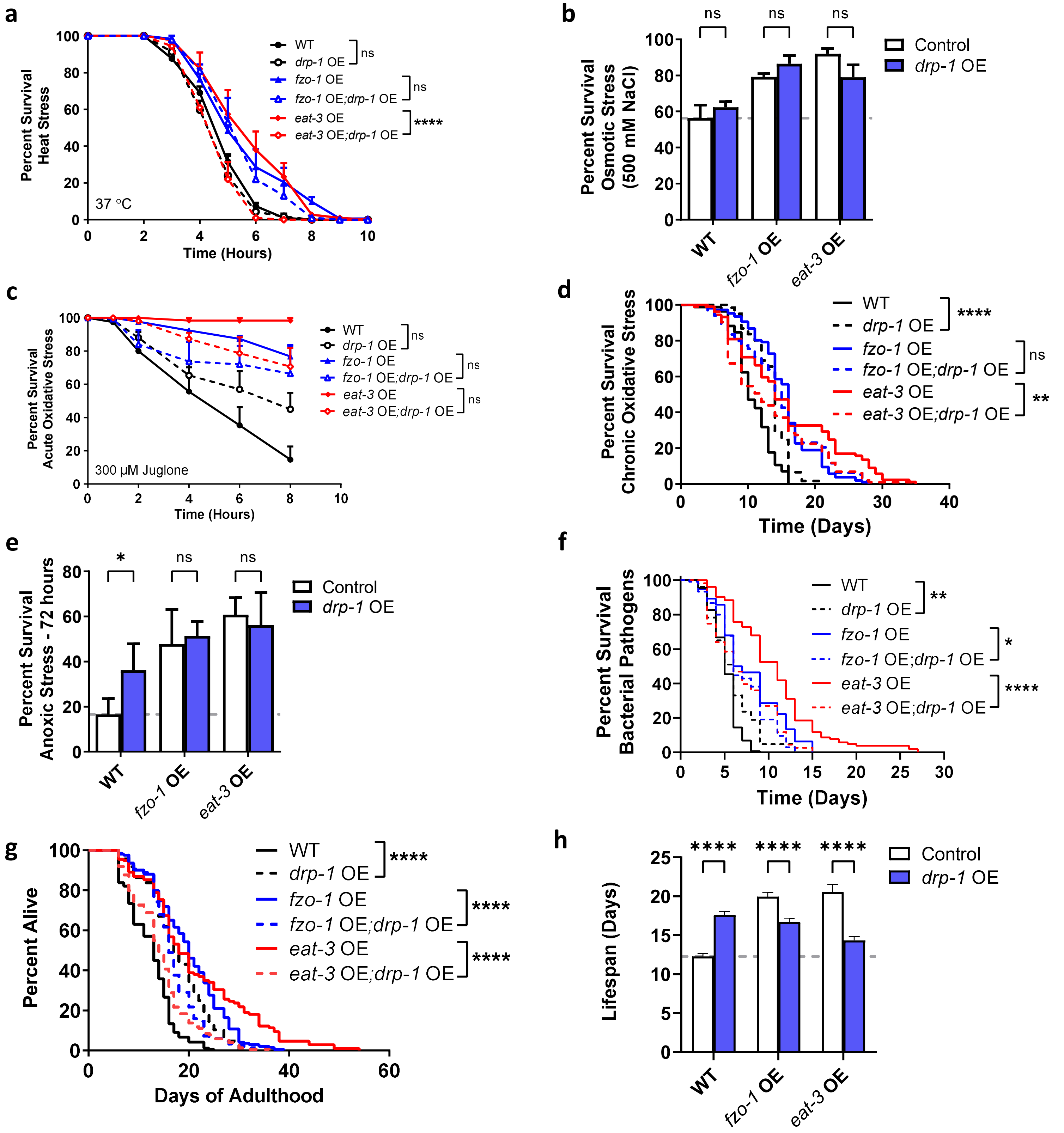

Figure S8. Overexpression *drp-1* decreases stress resistance and lifespan in worms overexpressing *eat-3*. (**a**) Overexpression of *drp-1* reverts heat stress resistance to wild-type in *eat-3* OE worms. (**b**) *eat-3* OE;*drp-1* OE worms exhibit a trend toward decreased osmotic stress resistance compared to *eat-3* OE worms. (**c**) *drp-1* OE decreases acute oxidative stress resistance in *fzo-1* OE and *eat-3* OE worms. (**d**) *drp-1* OE also reduces resistance to chronic oxidative stress in *eat-3* OE worms. (**e**) Overexpression of *drp-1* does not affect anoxia resistance in *fzo-1* OE or *eat-3* OE worms. While *drp-1* OE increases bacterial pathogen resistance (**f**) and lifespan (**g,h**) in wild-type worms, it decreases resistance to bacterial pathogens and lifespan in worms overexpressing mitochondrial fusion genes. Error bars indicate SEM. A minimum of three biological replicates were performed. Statistical significance was assessed using a repeated measures ANOVA with Tukey's multiple comparisons test in a and c; a two-way ANOVA with Šidák's multiple comparisons test in b, e, and h; and a log-rank test in d, f and g. p-values indicate difference between control and *drp-1* OE. OE = overexpression. Data on single overexpression strains is also shown in Figures 3 and 4. The data on the single overexpression strains shown here was collected in the same experiment with double overexpression strains. *p<0.05, **p<0.01, ***p<0.001, ****p<0.0001.

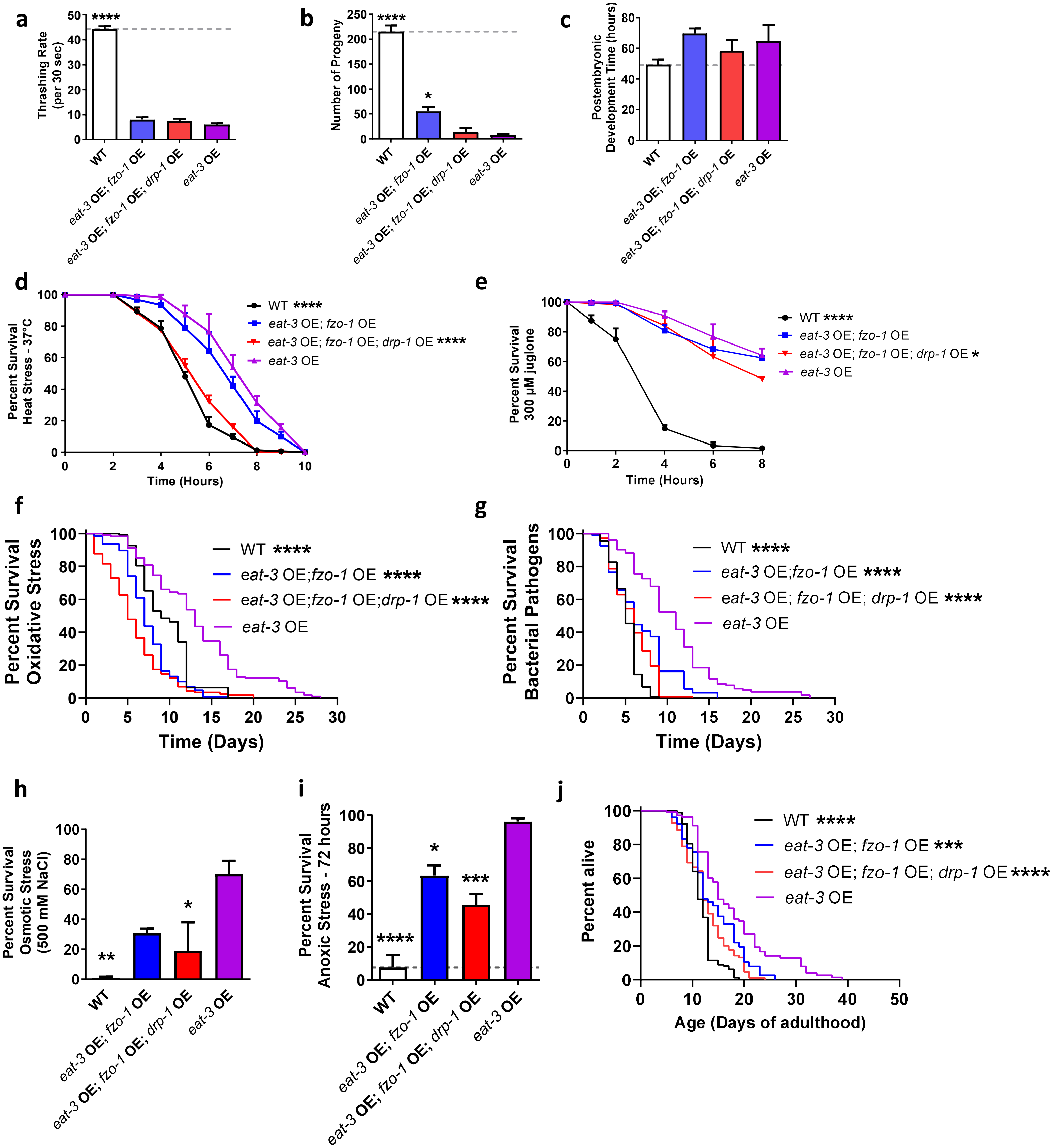

Figure S9. Effects of overexpressing *drp-1, fzo-1* and *eat-3* are not additive.

Full figure legend on following page.

Figure S9. Effects of overexpressing *drp-1, fzo-1* and *eat-3* are not additive. To examine the effects of increasing the expression of *drp-1, fzo-1* and *eat-3* simultaneously, we generated *eat-3* OE; *fzo-1* OE; *drp-1* OE worms. Similar to *eat-3* OE worms, *eat-3* OE; *fzo-1* OE; *drp-1* OE worms exhibit decreased movement (**a**), reduced fertility (**b**), and slow post-embryonic development (**c**). These worms have only a small increase in resistance to heat stress (**d**). While *eat-3* OE; *fzo-1* OE; *drp-1* OE worms have increased resistance to acute oxidative stress (**e**), they have increased sensitivity to chronic oxidative stress (**f**). *eat-3* OE; *fzo-1* OE; *drp-1* OE worms have a small increase in resistance to bacterial pathogens, which is less than observed in *eat-3* OE worms (**g**). These worms also exhibit a small increase in resistance to osmotic stress (**h**) and anoxia (**i**). Although *eat-3* OE; *fzo-1* OE; *drp-1* OE worms have increased lifespan compared to wild-type worms, the magnitude of increase is much less than in *eat-3* OE worms (**j**). Error bars indicate SEM. A minimum of three biological replicates were performed. Statistically significant differences from *eat-3* OE worms are indicated. Statistical significance was assessed using a one-way ANOVA with Dunnett's multiple comparisons test in a, b, c, h and i; a repeated measures ANOVA with Tukey's multiple comparisons test in d and e; and a log-rank test in f, g and j. p-values indicate statistical significance of differences from *eat-3* OE worms. OE = overexpression. Data on single *eat-3* overexpression strains is also shown in Figures 3 and 4. The data on the *eat-3* overexpression strains shown here was collected in the same experiment with double and triple overexpression strains. *p<0.05, **p<0.01, ***p<0.001, ****p<0.0001.

Table S1. Summary of phenotypes resulting from overexpression or deletion of mitochondrial fission and fusion genes. ↑ = increased, ↓ = decreased, "=" = unchanged, OE = overexpression. DEL = deletion. ND = not done. Data on deletion mutants is from Machiela et al. *FASEB J.* 2020.

	drp-1 OE	*fzo-1* OE	*eat-3* OE	*drp-1* DEL	*fzo-1* DEL	*eat-3* DEL
Mito number	↑	↑	↑	=	↑	↑
Mito. size	↓	↓	↓	=	↓	↓
Circularity	↑	↑	↑	=	↑	↑
Feret's diameter	↓	↓	↓	=	↓	↓
ATP levels (day 1)	=	=	↑	↓	↓	↓
Oxygen consumption (day 1)	=	=	↑	=	↓	↓
Movement (day 1)	↑	↑	↓	↓	=	↓
Fertility	↓	↓	↓	↓	↓	↓
Development time	=	↑	↑	↑	↑	↑
Heat stress	↑	↑	↑	↓	↑	↑
Osmotic stress	↑	↑	↑	↓	↓	↓
Acute oxidative stress	=	↑	↑	↑	↑	↑
Chronic oxidative stress	↑	↑	↑	↑	↑	↑
Anoxia	↑	↑	↑	↓	↓	↓
Bacterial pathogen stress	=	↑	↑	ND	ND	ND
Lifespan	↑	↑	↑	↑	=	↑
